# Impact of Urinalysis on Medical Decision-making and Length of Stay

**DOI:** 10.7759/cureus.2531

**Published:** 2018-04-25

**Authors:** Ambika Anand, Bethany Ballinger, Latha Ganti

**Affiliations:** 1 University of Central Florida College of Medicine, Orlando, USA; 2 Medical Education, University of Central Florida College of Medicine, Orlando, USA; 3 Clinical Sciences, University of Central Florida College of Medicine, Orlando, USA

**Keywords:** urinalysis, clinical decision-making, length of stay, emergency department, quality improvement

## Abstract

Introduction

The emergency department (ED) is under pressure to meet length of stay (LOS) metrics for care in the ED. An aspect that we propose affects LOS is the order for urine sample collection and subsequent urinalysis (UA) as both are time consuming steps. This project’s primary goals are to determine if ordering a UA increases LOS and how often UA contributes to clinical decision-making and/or disposition decisions in the ED. Secondary objectives were to identify factors that contribute to the ordering of a UA and to decipher if LOS was more impacted in patients who were discharged vs. admitted to the hospital.

Methods

Retrospective chart review was conducted of patients who presented to our ED in April 2016 during 12 consecutive days. Data were abstracted onto a data collection sheet with the abstractor blinded to study hypotheses. Variables included whether a UA was ordered, times of UA order and result, who ordered the UA (mid-level provider [MLP] vs. physician), whether the UA was cancelled, whether the UA result influenced clinical decision-making (based on the medical decision-making section of the physician chart) or disposition decision, LOS, age, and gender. Descriptive statistics and multivariable regression analysis were used to analyze relationships between the variables collected and their influence on LOS.

Results

The overall median LOS was 157 minutes, with an interquartile range (IQR) of 81 to 246 minutes. For discharged patients, it was 142 minutes, with an IQR of 46 to 236 minutes. For admitted patients, it was 177 minutes, with an IQR of 118 to 260 minutes. Amongst admitted patients, multivariable regression analysis demonstrated that the following factor was associated with increased LOS: being seen first by the provider-in-triage (PIT) then physician in main ED (p < 0.0001). Amongst discharged patients, multivariable regression analysis demonstrated that the following factors were associated with increased LOS: being seen first by the PIT then physician in main ED (p = 0.0296), being seen by MLP only (p < 0.0001), having a UA ordered (p = 0.0005), being seen on weekend (p = 0.0166), and being an older patient (p = 0.0475). The UA was cancelled in 9% of our patients, and in 60% of cases, these UAs were ordered by the PIT. Patient disposition decision was made prior to UA resulting in 60 cases (25%). The UA was used in clinical decision-making in 118 cases (66%). The following predictor factors were associated via univariate analysis with using a UA for decision-making: being female (p = 0.0050, 95% CI: 0.0068–0.378), being an older patient (p < 0.0001, 95% CI: -0.010 to -0.004), being first seen by the PIT and then a physician (p = 0.0486, 95% CI: 0.0048–0.1555), and discharged patients (p < 0.0001, 95% CI: -0.6749 to -0.4487).

Conclusion

Our results suggest that having a UA ordered increased ED LOS, especially in patients who are ultimately discharged. In our ED, routine UAs are ordered more often by MLPs than physicians. A routine UA may not impact clinical decision-making up to 33% of the time, nor alter disposition decision one out of four times. Given that 9% have the test eventually cancelled, one should reconsider the utility in ordering routine UAs in ED patients, as they increase LOS and place an additional burden on the patient and the ED personnel.

## Introduction

The emergency department (ED) increasingly must balance meeting metrics for time and quality with patient satisfaction. The ED is at the foreground of innovation for increasing efficiency and patient throughput without losing quality. Over the past few years, studies have focused on the quality of care when implementing a time limit [1], methods of improving ED flow and decreasing key metrics such as length of stay (LOS), left without treatment (LWOT) [2], time-to-provider metrics [3,4], and the benefits of regular reporting and regulation of metrics [5]. In addition, the effect of laboratory testing and imaging on LOS has been studied in terms of turnaround time and test volume, and increased numbers have been found to correlate directly with increased LOS [6,7]. Specifically, urine collection and urinalysis (UA) are two aspects suspected to take a significant amount of time, both in the collection process of a urine sample from a patient and in the time it takes for a urinalysis result to return [7].

There have been instances in which ED providers order urinalysis on a patient that presents without symptoms that warrant the testing. This can happen when considering a urinary tract infection vs. asymptomatic bacteriuria, two conditions that require different methods of treatment in terms of antibiotics [8-10].

This study intends to determine if LOS increases for patients that have a UA ordered, how often UAs are ordered in the ED, how often the UA result affects patient management, how often the UA results are not used in the medical decision-making process, and how often a UA is ordered and results do not return before disposition, therefore making the time spent collecting and analyzing unnecessary. Lab testing is frequently conducted in the ED, and this project intends to determine how often a UA is worth the length of stay it costs. A future goal of the project is to create and implement a decisional algorithm to increase patient specificity for urine tests and urine collection at triage and determine if doing so will affect LOS of patients that require urine samples. It is predicted that implementing an algorithm will decrease LOS of patients that require a urinalysis and will also decrease the number of urinalyses that are ordered on patients that reach disposition without results returning.

The significance of this project lay in its ability to working towards a method to decrease LOS and improve ED metrics and patient flow through analyzing markers associated with UA orders. It additionally has the potential to decrease the number of unnecessary urine samples collected or UAs completed to improve resource efficiency. The project also takes a detailed look at the way emergency physicians order and use UA results in their decision-making process in the ED.

## Materials and methods

This was a retrospective chart review of all patients who presented to our ED between the dates of April 16, 2016 and April 27, 2016. Our institution is a suburban ED with over 80,000 visits per year and is home to an emergency medicine residency program. Our medical school’s Institutional Review Board approved the study with a waiver of informed consent. JMP 12.0 for Mac OS X was used to conduct statistical analysis.

The acquired data included the following: patient LOS, UA orders, whether patient management was influenced by UA results as per medical decision making and other documentation versus the number in which they were not used, whether a UA was ordered but results were not obtained prior to disposition, disposition decisions, UA collection and result times, UA order cancellations, the provider that ordered the UA (mid-level provider (MLP) vs. physicians), gender, time of presentation, weekend or weekday presentation, and age. Variables were chosen based on clinical expertise.

Data collection was conducted by obtaining a master list of patient names of those that were seen in the ED during the specified time period and manually conducting chart reviews for the variables stated above.

Demographic variables (gender, age, time of presentation, and weekday or weekend presentation) are described using descriptive statistics (mean, median, mode, interquartile range (IQR)). Multivariable regression analysis was used to establish associations between the variables collected. Specifically, associations were drawn between the variables and UA orders or LOS.

## Results

The cohort consisted of 559 patients, of which 368 (66%) were female. The median age of all patients was 51 with an IQR of 35 to 68 years. A total of 294 patients (38%) presented on a weekend, which was defined as Friday 7 pm to Monday 7 am; 101 patients (35%) presented during the night shift, which was defined as arriving between the hours of 7 pm and 7 am. A total of 265 patients (65%) were seen by an MLP in triage (noted as provider-in-triage or PIT); 138 patients (25%) were seen by an MLP after triage in the ED. A total of 232 patients (42%) were admitted.

Of the total number of patients, 287 patients (51%) had a UA ordered while in the ED, whereas 193 patients (34% of all patients) had their UA ordered by the PIT. Ninety-four patients (17% of all patients) had their UA ordered by the physician. The UA order was cancelled in 50 patients (9%) of all patients.

The overall median LOS was 157 minutes, with an IQR of 81 to 246 minutes. For discharged patients, the median LOS was 142 minutes, with an IQR of 46 to 236 minutes. For admitted patients, the median LOS was 177 minutes, with an IQR of 118 to 260 minutes.

Multivariable regression analysis was conducted to determine variables associated with an increased LOS amongst all patients (admitted and discharged), just admitted patients, and just discharged patients. Findings are presented in Table 1.

**Table 1:** Three multivariate regression analyses predicting LOS. Nonsignificant predictor factors (not listed) were: time of presentation (night shift or day shift) and gender. LOS: Length of stay; PIT: Mid-level provider in triage; MLP: Mid-level provider; UA: Urinalysis.

Predictor factors	All patients (n = 559)	Admitted patients (n = 232)	Discharged patients (n = 327)
PIT	p < 0.0001	p < 0.0001	p = 0.0296
Seen by MLP	p < 0.0001	ns	p < 0.0001
UA order	p < 0.0001	ns	p = 0.0005
Seen on a weekend	p < 0.001	ns	p = 0.0166
Older age	ns	ns	p = 0.0475

In a multivariable regression model looking at who had a UA ordered, the most statistically significant variable for having a UA ordered was having been seen by the PIT (p = 0.0017, 95% CI: 0.0766–0.3270). Age, gender, weekend presentation, and night shift presentation were not statistically significant.

The disposition decision was made prior to the UA result returning for 60 patients (25% of patients who had a UA order that was not cancelled). Of those patients, 36 patients (60%) were women. The median age was 65, with an IQR of 49 to 73. Twenty-nine patients (48%) were seen on the weekend, though the days from which the data was collected included four weekend days out of 12 total days. Twenty patients (33%) were seen on the night shift. Fifty-six patients (93%) were not seen by the PIT. Eighteen patients (30%) had their UA ordered by the PIT or the MLP in the main ED, and 42 patients (70%) had their UA ordered by the physician. Of these 60 patients, 50 had their UA cancelled.

Characteristics of the 50 patients with a cancelled UA order were as follows: 33 patients (66% of patients with the cancelled UA order) were women; the median age was 50, with an IQR of 37 to 65; median LOS was 186 minutes, with an IQR of 115 to 250 minutes; 19 patients (38%) were seen on the weekend; 15 patients (30%) were seen on night shift; 32 patients (64%) were seen by the PIT; 30 patients (60%) had their UA ordered by the MLP in triage; eight patients (16%) were seen by an MLP after triage; 18 patients (36%) were admitted; and 20 patients (40%) had their UA ordered by the provider (MLP or physician).

The UA result was used for medical decision-making for 118 patients (66%) that had a UA ordered and had the UA result return before the disposition decision was made. Univariate regression analysis indicated that the following predictor factors listed in Table 2 were associated with a UA result being used for clinical decision-making.

**Table 2:** A univariate regression analysis predicting if the UA was used for decision-making. Nonsignificant predictor factors (not listed) were: time of presentation (night shift or day shift), day of presentation (weekend or weekday), and if they were seen by the MLP. UA: Urinalysis; PIT: Mid-level provider in triage; MLP: Mid-level provider.

Predictor factor	UA used for decision-making (n = 118)
Female	p = 0.0050 (95% CI: 0.068–0.378)
Older age	p < 0.0001 (95% CI: -0.010 to -0.004)
PIT	p = 0.0486 (95% CI: 0.0048–0.1555)
Discharged patient	p < 0.0001 (95% CI: -0.6749 to -0.4487)

## Discussion

As the UA order is often placed in a reflexive manner, its role in clinical decision-making was under investigation in this project. The majority of patients (51%) had a UA ordered placed during their visit in the ED. Moreover, routine UAs were more likely to be ordered by an MLP than a physician. Having a UA ordered increased the LOS, especially in patients that were ultimately discharged. Considering that 17% of all UAs ordered were cancelled, the utility of a UA should be reconsidered in order to best optimize the LOS metric and alleviate the burden for patients and ED personnel.

The only predictor factor associated with increased LOS across the three multivariate regression analyses involving LOS was evaluation by the PIT. Though the PIT program's goal is to reduce LOS and increase ED efficiency through early evaluation, it may have played a role in increasing LOS for patients. Alternatively, it may be that the patients with a longer LOS would naturally have a longer stay in the ED due to underlying co-morbidities. Regardless, this pattern indicates that it may be warranted to continue further with this project to the eventual goal of creating a decisional algorithm that may have the greatest utility in the triage step of the ED visit. An algorithm has the possibility of decreasing the number of UAs ordered and better specifying which patients may require a UA.

Of the patients that had a UA ordered that was not cancelled, 25% had their disposition decision made prior to the UA result returning, suggesting that the result of the routine UA would not alter the disposition decision up to a quarter of the time. In patients that did have a UA result return prior to making the disposition decision, the UA result did not impact medical decision-making up to 33% of the time. This indicates that the routine UA may not have been a necessary order for the patient. This suggests that UAs may be an unnecessary routine test, and their role in protocols should be reevaluated.

Technical limitations related to data storage impeded access to certain patient charts. Per the method of data storage used by the Hospital Corporations of America (HCA), some charts were unavailable for review despite gaining Privacy Approval by the hospital’s Privacy Officer. This limited the number of charts available for review and may have skewed the cohort that appears to have presented to the ED. In addition, this ED’s flow included an MLP in triage before being seen by another MLP or a physician. Not all EDs have this particular structure for a patient visit, so it may be difficult to extrapolate results to those dissimilar ED flows.

Future work should consider further evaluation of factors that lead to a UA order, such as acuity level, and methods of streamlining the process to collect and analyze a UA.

## Conclusions

This project demonstrated an association between UA orders and increased LOS and found that they were more likely to be ordered by an MLPs than physicians. UAs may not impact medical decision making up to 33% of the time and may not be influential for disposition decisions up to 25% of the time. These results indicate that the utility of a UA should be reconsidered in order to best optimize the LOS metric and alleviate the burden for patients and ED personnel. In future subsequent steps, it is expected that this data will be used to create a decisional algorithm in the future for emergency physicians and ED staff to follow when triaging patients that would potentially require a UA.
